# Supplementary material for: Absent in Melanoma 2 (AIM2) Regulates the Stability of Regulatory T Cells
Source: Int J Mol Sci. 2022 Feb 17;23(4):2230. doi: 10.3390/ijms23042230 (PMC8876789; doi:10.3390/ijms23042230)
Supplement: Supplementary file 1 [file ijms-23-02230-s001.zip › ijms-1405637-supplementary/ijms_1405637_Suppl Fig_revised.pdf]

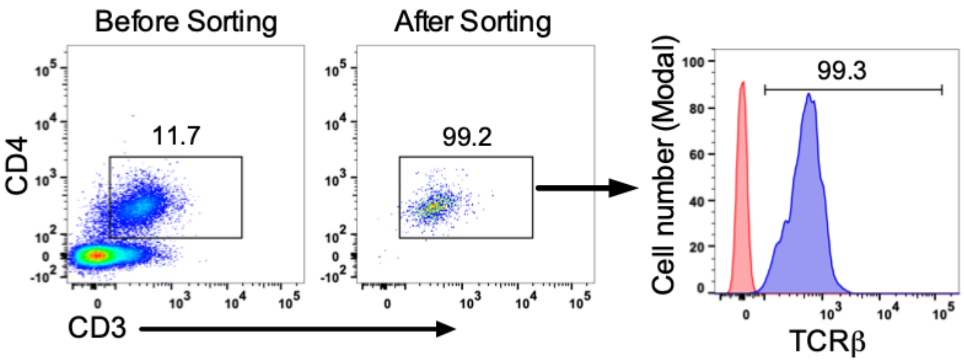

Figure S1. Purity of the sorted CD4+ cells.

Flow cytometry analysis of the expression of CD3+CD4+TCRβ+ cells after sorting in a FACSAria III flow cytometer using FACSDiva software. Purity of the sorted populations was higher than 98.5% in all experiments.

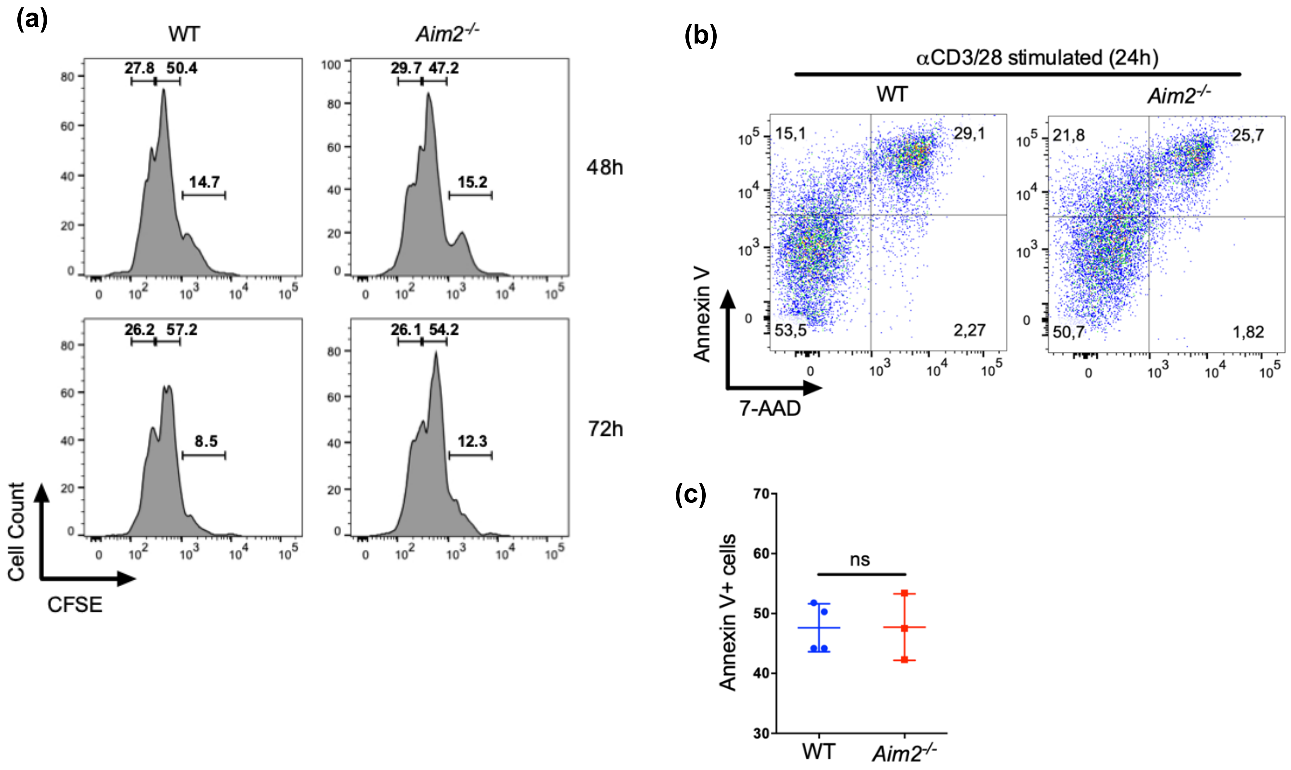

Figure S2. Activation-induced cell death and proliferation in WT and *Aim2*$^{-/-}$ CD4^{+} T cells.
A) Evaluation of proliferative cells by flow cytometry analysis of CFSE-labeled CD4^{+} T cells from WT or *Aim2*$^{-/-}$ mice after 48 and 72 hours of anti-CD3/CD28 stimulation. Data are from one mouse, representative of 4 mice per group. **B)** Flow cytometry analysis of early apoptotic (Annexin V+, 7-AAD-), late-stage apoptotic (Annexin V+, 7-AAD+), and necrotic (Annexin V-, 7-AAD+) cells from WT (n=4) or *Aim2*$^{-/-}$ (n=3) mice after 24 hours of stimulation with anti-CD3/CD28 antibodies. **C)** Scatter plot graph of the percentage of total Annexin V positive cells. Data were analyzed using t test with Welch's correction, ns = not significant.

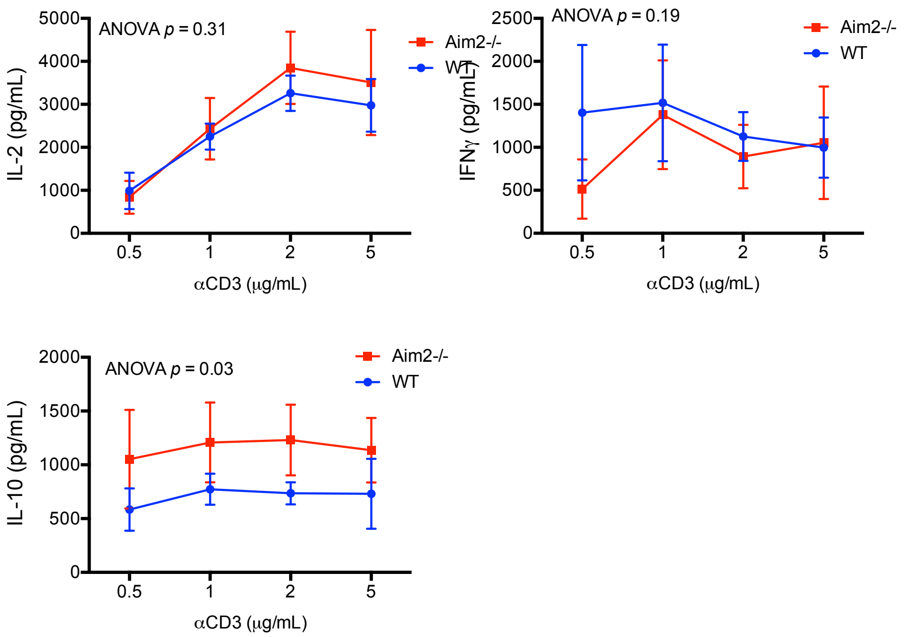

Figure S3. Stimulation threshold in WT and *Aim2*$^{-/-}$ CD4^{+} T cells

Splenic CD4^{+} T cells from WT or *Aim2*$^{-/-}$ mice (n=3 per group) were stimulated with increasing concentrations of plate-bound anti-CD3 antibody (αCD3) and a fixed concentration of anti-CD28 antibody (2 µg/mL). Culture supernatants were collected at 24 hours and analyzed for IL-2, IFNγ, and IL-10 production by ELISA. Results were analyzed by two-way ANOVA.

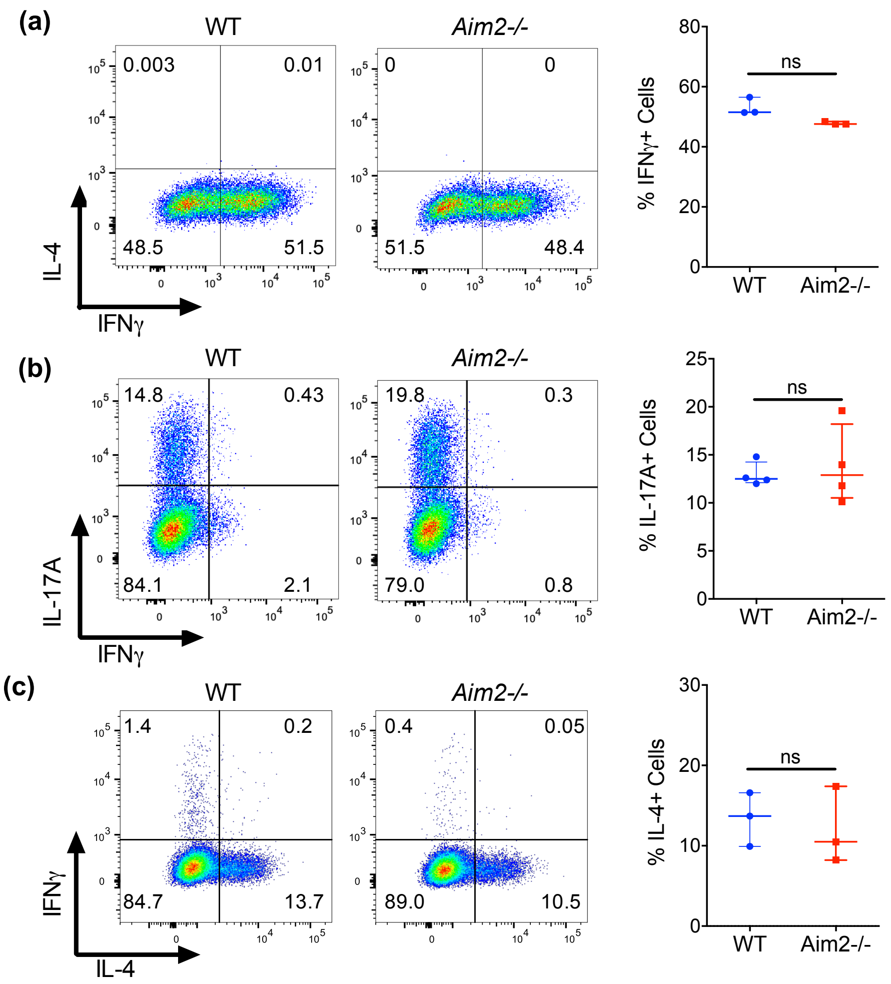

Figure S4. AIM2 deficiency does not affect Th1, Th2 or Th17 differentiation
(a-c) Flow cytometry analysis of intracellular cytokines in naïve CD4+ T cells from WT or *Aim2*-/- mice cultured in Th1 (a), Th2 (b) or Th17 (c) polarizing conditions for 5 days and re-stimulated with anti-CD3/CD28 antibodies for 5 hours. Data were analyzed using t test with Welch's correction, and are displayed as scatter plot with median ± interquartile range, ns = not significant.

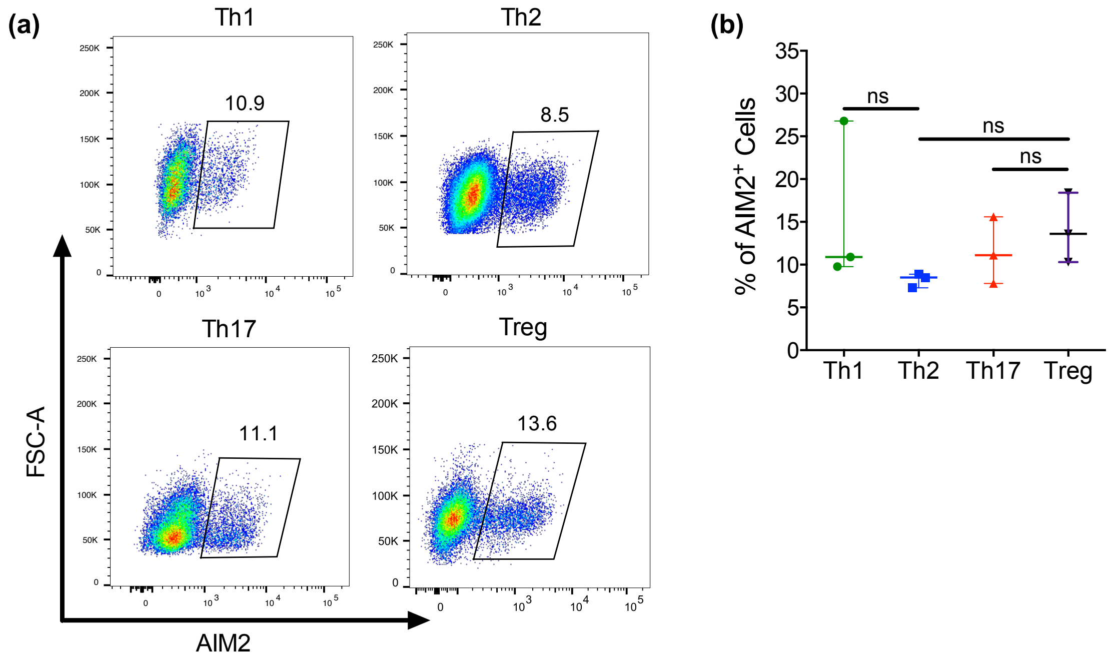

Figure S5. AIM2 expression in different CD4+ T cell subsets generated in vitro
(a) Flow cytometry analysis of AIM2 expression in WT naïve CD4+ T cells 5 days after culture in Th1, Th2, Th17 or Treg (iTreg) conditions. **(b)** Data from three mice in each condition were analyzed using a Holm-Sidak's multiple comparisons test and displayed as scatter plot with median ± interquartile range, ns = not significant.

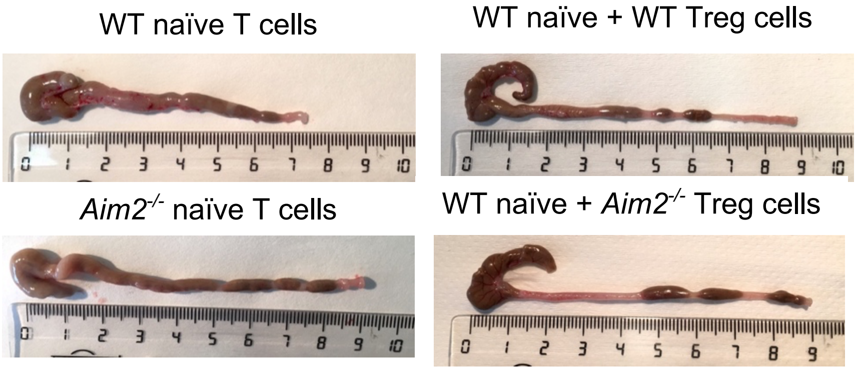

Figure S6. Macroscopic analysis of colons from *Rag1*$^{-/-}$ mice after adoptive transfer
Representative pictures of colons from *Rag1*$^{-/-}$ mice reconstituted with naïve T cells ($CD4^{+}CD45RB^{high}CD25^{-}$) from WT or *Aim2*$^{-/-}$ mice, or cotransferred with WT naïve T cells plus regulatory T cells ($CD4^{+}CD45RB^{low}CD25^{+}$) from either WT or *Aim2*$^{-/-}$ mice.

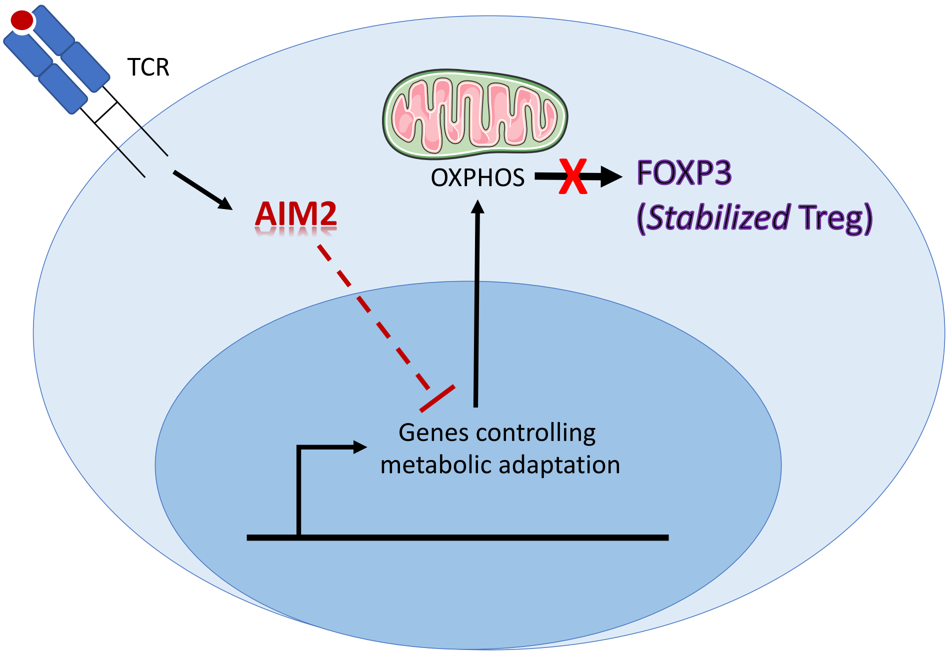

Figure S7. Graphical abstract.
Upon T cell receptor (TCR) activation of CD4⁺ T cells, AIM2 expression is transiently increased and suppresses the expression of genes associated with oxidative phosphorylation (OXPHOS) and mitochondrial respiration, thereby reducing FOXP3 expression and Treg stability.
