# Supplementary material for: Absent in Melanoma 2 (AIM2) Regulates the Stability of Regulatory T Cells
Source: Int J Mol Sci. 2022 Feb 17;23(4):2230. doi: 10.3390/ijms23042230 (PMC8876789; doi:10.3390/ijms23042230)
Supplement: Supplementary file 1 [file ijms-23-02230-s001.zip › ijms-1405637-supplementary/ijms_1405637_Suppl Table.pdf]

Table S1. List of reagents, antibodies and primers

1.1. Reagents.

Product Name	Supplier	Catalog no.
**EasySep™ Mouse CD4+ T Cell Isolation Kit**	STEMCELL Technologies, Vancouver, Canada	19852
**EasySep™ Mouse Naïve CD4+ T Cell Isolation Kit**	STEMCELL Technologies, Vancouver, Canada	19765
**Intracellular Fixation and Permeabilization Buffer + Brefeldin A**	eBioscience, San Diego, CA, USA	88-8823-88
**H_2O_2 30%**	Sigma Aldrich, St. Louis, MO, USA	H-1009
**Bovine Serum Albumine (BSA)**	Santa Cruz Biotechnologies	sc-2323
**L-Lysine monohydrochloride**	Sigma Aldrich, St. Louis, MO, USA	L5626-500G
**Endogenous Avidin + Biotin blocking solution**	abcam, Cambridge, United Kingdom	ab64212
**Vectastain ABC-HRP kit, Peroxidase (Standard)**	Vector Laboratories, Burlingame, CA, USA	PK-4000
**DAB peroxidase substrate kit**	Vector Laboratories, Burlingame, CA, USA	SK-4100
**Eukitt® mounting medium**	Panreac, Barcelona, Spain	253681
**Biocoll Separating Solution**	Merck, Kenilworth, New Jersey, USA	L6115
**EasySep™ Human CD4+ T cell isolation kit**	STEMCELL Technologies, Vancouver, USA	17952
**Advanced RPMI 1640 medium**	Life Technologies, Carlsbad, CA, USA	12633020
**IMDM medium with L-Glutamin (4mM)**	Life Technologies, Carlsbad, CA, USA	21980032
**Fetal Bovine Serum, heat inactivated**	Life Technologies, Carlsbad, CA, USA	10082-147
**L-Glutamine (200 mM)**	Life Technologies, Carlsbad, CA, USA	25030-024
**Penicillin-Streptomycin (10000 U/mL)**	Life Technologies, Carlsbad, CA, USA	15140-122
**IL17A mouse uncoated ELISA Ready-Set-Go kit**	Life Technologies, Carlsbad, CA, USA	88-7371-88
**IL10 mouse uncoated ELISA Ready-Set-Go kit**	Life Technologies, Carlsbad, CA, USA	88-7105-77
**IL4 mouse uncoated ELISA Ready-Set-Go kit**	Life Technologies, Carlsbad, CA, USA	88-7044-88
**IFNg mouse uncoated ELISA Ready-Set-Go kit**	Life Technologies, Carlsbad, CA, USA	88-7314-88
**Recombinant mouse IL-12p70 protein**	Peprotech, Rocky Hill, NJ, USA	AF-210-12
**Recombinant mouse IL-2 protein**	Peprotech, Rocky Hill, NJ, USA	AF-212-12
**Recombinant mouse IL-6 protein**	Peprotech, Rocky Hill, NJ, USA	AF-216-16
**Recombinant human TGF-b protein**	Peprotech, Rocky Hill, NJ, USA	AF-100-21
**Recombinant mouse IL-4 protein**	R&D Systems, Minneapolis,USA	404-ML-010

**RNeasy Mini kit**	Qiagen, Hilden, Germany	74106
**qScript One-step RT-qPCR kit**	QuantaBio, Beverly, USA	QUNT95086-200
**RNAlater**	Qiagen, Hilden, Germany	76106
**Protease inhibitor cocktail**	Sigma Aldrich, St. Louis, MO, USA	P8340
**LDS sample buffer (4X) NuPAGE**	Thermo Fisher Scientific, Waltham, USA	NP0008
**β-2mercaptoethanol**	Thermo Fisher Scientific, Waltham, USA	31350010
**NuPAGE 4-12% Bis-Tris, 1.0 mm Mini Protein Gel, 10-wells**	Thermo Fisher Scientific, Waltham, USA	NW04122BOX
**Immunoblot PVDF membranes**	BioRad, Hercules, CA, USA	1620177
**Tween 20**	Merck, Kenilworth, New Jersy, USA	P9416
**Supersignal West Femto Chemi**	Thermo Fisher Scientific, Waltham, USA	W6421N
**Supersignal West Pico Chemi**	Thermo Fisher Scientific, Waltham, USA	34080
**CFSE**	Labclinics, Barcelona, Spain	65-0850--84
**CellTrace Far Red Cell Proliferation Kit**	Thermo Fisher Scientific, Waltham, USA	C34564
**Annexin V**	Labclinics, Barcelona, Spain	88-8102-72
**7-AAD Viability Staining Solution**	Labclinics, Barcelona, Spain	00-6993-50
**Formalin Solution Neutral Buffered, 10%**	Sigma Aldrich, St. Louis, MO, USA	HT501128
**GemCut Paraffin, 56-58ºC**	Polysciences, Warrington, PA, USA	24361-1
**Harris Haematoxylin**	Leica Biosystems, Wetzlar, Germany	3801561
**Eosin-Phloxin**	DiaPath, Martinengo, Italy	C0343
**Dynabeads™ Mouse T-Activator CD3/CD28 for T-Cell Expansion and Activation**	Life Technologies, Carlsbad, CA, USA	11456D
**Qubit RNA HS Assay Kit**	Thermo Fisher Scientific, Waltham, USA	Q32855
**Agilent RNA 6000 Nano Chips**	Agilent Technologies	5067-1511
**TruSeq Stranded mRNA Library Prep Kit set A**	Illumina	RS-122-2101
**TruSeq Stranded mRNA Library Prep Kit set B**	Illumina	RS-122-2102
**DNA-free DNA removal kit**	Thermo Fisher Scientific, Waltham, USA	AM1906

1.2. Antibodies.

**Name**	**Supplier**	**Catalog no.**	**Clone no.**
**Anti mouse CD3 antibody,**	eBioscience – ThermoFisher	16-0031-86	17A2

**functional grade purified**	Scientific, Waltham, MA, USA		
**Anti mouse CD28 antibody, functional grade purified**	eBioscience – ThermoFisher Scientific, Waltham, MA, USA	16-0281-82	37.51
**IL-12 p35 Rat anti-Mouse**	Ivitrogen – ThermoFisher Scientific, Waltham, MA, USA	14-7122-285	C18.2
**IL-4 Rat anti-Mouse**	Ivitrogen – ThermoFisher Scientific, Waltham, MA, USA	16-7041-85	11B11
**Anti-FOXP3 Monoclonal Antibody**	eBioscience – ThermoFisher Scientific, Waltham, MA, USA	14-5773-82	FJK-16s
**Biotinylated goat anti-Rat IgG**	Vector Laboratories, Burlingame, CA, USA	BA-9400	Polyclonal
**In Vivo mAb Anti-mouse IL4 antibody**	BioxCell, Lebanon, NH, USA	BE0045	11B11
**In Vivo mAb Anti-mouse IFNg antibody**	BioxCell, Lebanon, NH, USA	BE0055	XMG1.2
**IL17 antibody, anti-mouse, FITC**	Milteny Biotec, Bergisch Gladbach, Germany	130-102-262	TC11-18H10
**IFNg antibody, anti-mouse, APC**	Milteny Biotec, Bergisch Gladbach, Germany	130-109-723	REA638
**FOXP3 antibody, anti-mouse, PE**	Milteny Biotec, Bergisch Gladbach, Germany	130-111-678	REA788
**AIM2 Polyclonal Antibody**	eBioscience – ThermoFisher Scientific, Waltham, MA, USA	14-6008-93	n/a
**Goat anti-Rabbit IgG, Secondary Antibody, Alexa Fluor 647**	Ivitrogen – ThermoFisher Scientific, Waltham, MA, USA	A21244	n/a
**Rat IgG1 isotype control antibody**	Milteny Biotec, Bergisch Gladbach, Germany	130-102-644	ES26-14D1.11
**REA control antibody, APC**	Milteny Biotec, Bergisch Gladbach, Germany	130-104-630	n/a
**REA control antibody, PE**	Milteny Biotec, Bergisch Gladbach, Germany	130-104-628	n/a
**AIM2 (D-14) antibody**	Santa Cruz Biotechnologies, Dallas, Texas, USA	sc-137967	Polyclonal
**Anti-Rabbit IgG HRP secondary antibody**	Jackson Immunoresearch, West Grove, PA, USA	111-035-003	Polyclonal
**ASC (N-15) antibody**	Santa Cruz Biotechnologies, Dallas, Texas, USA	sc-22514-R	Polyclonal
**CASP-1 14F468 antibody**	Santa Cruz Biotechnologies, Dallas, Texas, USA	sc-56036	14F468
**β-actin antibody**	Merck, Kenilworth, New Jersey, USA	A1978	AC-15
**Anti-Mouse IgG HRP secondary antibody**	Jackson Immunoresearch, West Grove, PA, USA	115-035-003	Polyclonal

1.3. Primers. All primers were purchased from Integrated DNA Technologies (Coralville, IA, USA).

Name	Sequence
**Aim2 forward primer for qPCR**	5'-TGGCCACGGAGACAGATTTT-3'
**Aim2 reverse primer for qPCR**	5'-GGAACTGGTGACCTCCATGA-3'
**Nlrp3 forward primer for qPCR**	5'-CCGTCTACGTCTTCTTCCTTTC -3'
**Nlrp3 reverse primer for qPCR**	5'-CGCAGATCACACTCCTCAAATA -3'
**Casp1 forward primer for qPCR**	5'-TACACGTCTTGCCCTCATTATC-3'
**Casp1 reverse primer for qPCR**	5'-CTCCAGCAGCAACTTCATTTC-3'
**Gapdh forward primer for qPCR**	5’-CTTTGGCATTGTGGAAGGGC-3’
**Gapdh reverse primer for qPCR**	5’-CAGGGATGATGTTCTGGGCA-3’
